# Predicting the number of oocytes retrieved from controlled ovarian hyperstimulation with machine learning

**DOI:** 10.1093/humrep/dead163

**Published:** 2023-08-15

**Authors:** Timothy Ferrand, Justine Boulant, Chloe He, Jérôme Chambost, Céline Jacques, Chris-Alexandre Pena, Cristina Hickman, Arnaud Reignier, Thomas Fréour

**Affiliations:** AI Team, Apricity, Paris, France; Centre Hospitalier Universitaire de Nantes, Nantes, France; AI Team, Apricity, London, UK; Wellcome/EPSRC Centre for Interventional and Surgical Sciences, University College London, London, UK; Department of Computer Science, University College London, London, UK; AI Team, Apricity, Paris, France; AI Team, Apricity, Paris, France; AI Team, Apricity, Paris, France; AI Team, Apricity, London, UK; Institute of Reproductive and Developmental Biology, Imperial College London, London, UK; Centre Hospitalier Universitaire de Nantes, Nantes, France; Centre Hospitalier Universitaire de Nantes, Nantes, France; Department of Reproductive Medicine, Dexeus University Hospital, Barcelona, Spain

**Keywords:** ovarian stimulation, oocyte retrieval, machine learning, artificial intelligence, trustworthy machine learning, predictive modelling

## Abstract

**STUDY QUESTION:**

Can machine learning predict the number of oocytes retrieved from controlled ovarian hyperstimulation (COH)?

**SUMMARY ANSWER:**

Three machine-learning models were successfully trained to predict the number of oocytes retrieved from COH.

**WHAT IS KNOWN ALREADY:**

A number of previous studies have identified and built predictive models on factors that influence the number of oocytes retrieved during COH. Many of these studies are, however, limited in the fact that they only consider a small number of variables in isolation.

**STUDY DESIGN, SIZE, DURATION:**

This study was a retrospective analysis of a dataset of 11,286 cycles performed at a single centre in France between 2009 and 2020 with the aim of building a predictive model for the number of oocytes retrieved from ovarian stimulation. The analysis was carried out by a data analysis team external to the centre using the Substra framework. The Substra framework enabled the data analysis team to send computer code to run securely on the centre’s on-premises server. In this way, a high level of data security was achieved as the data analysis team did not have direct access to the data, nor did the data leave the centre at any point during the study.

**PARTICIPANTS/MATERIALS, SETTING, METHODS:**

The Light Gradient Boosting Machine algorithm was used to produce three predictive models: one that directly predicted the number of oocytes retrieved and two that predicted which of a set of bins provided by two clinicians the number of oocytes retrieved fell into. The resulting models were evaluated on a held-out test set and compared to linear and logistic regression baselines. In addition, the models themselves were analysed to identify the parameters that had the biggest impact on their predictions.

**MAIN RESULTS AND THE ROLE OF CHANCE:**

On average, the model that directly predicted the number of oocytes retrieved deviated from the ground truth by 4.21 oocytes. The model that predicted the first clinician’s bins deviated by 0.73 bins whereas the model for the second clinician deviated by 0.62 bins. For all models, performance was best within the first and third quartiles of the target variable, with the model underpredicting extreme values of the target variable (no oocytes and large numbers of oocytes retrieved). Nevertheless, the erroneous predictions made for these extreme cases were still within the vicinity of the true value. Overall, all three models agreed on the importance of each feature which was estimated using Shapley Additive Explanation (SHAP) values. The feature with the highest mean absolute SHAP value (and thus the highest importance) was the antral follicle count, followed by basal AMH and FSH. Of the other hormonal features, basal TSH, LH, and testosterone levels were similarly important and baseline LH was the least important. The treatment characteristic with the highest SHAP value was the initial dose of gonadotropins.

**LIMITATIONS, REASONS FOR CAUTION:**

The models produced in this study were trained on a cohort from a single centre. They should thus not be used in clinical practice until trained and evaluated on a larger cohort more representative of the general population.

**WIDER IMPLICATIONS OF FINDINGS:**

These predictive models for the number of oocytes retrieved from COH may be useful in clinical practice, assisting clinicians in optimizing COH protocols for individual patients. Our work also demonstrates the promise of using the Substra framework for allowing external researchers to provide clinically relevant insights on sensitive fertility data in a fully secure, trustworthy manner and opens a number of exciting avenues for accelerating future research.

**STUDY FUNDING/COMPETING INTEREST(S):**

This study was funded by the French Public Bank of Investment as part of the Healthchain Consortium. T.Fe., C.He., J.C., C.J., C.-A.P., and C.Hi. are employed by Apricity. C.Hi. has received consulting fees and honoraria from Vitrolife, Merck Serono, Ferring, Cooper Surgical, Dibimed, Apricity, and Fairtility and travel support from Fairtility and Vitrolife, participates on an advisory board for Merck Serono, was the founder and organizer of the AI Fertility conference, has stock in Aria Fertility, TMRW, Fairtility, Apricity, and IVF Professionals, and received free equipment from Planar in exchange for first user feedback. C.J. has received a grant from BPI. J.C. has also received a grant from BPI, is a member of the Merck AI advisory board, and is a board member of Labelia Labs. C.He has a contract for medical writing of this manuscript by CHU Nantes and has received travel support from Apricity. A.R. haș received honoraria from Ferring and Organon. T.Fe. has received a grant from BPI.

**TRIAL REGISTRATION NUMBER:**

N/A.

## Introduction

An integral and critical stage in IVF cycles is that of controlled ovarian hyperstimulation (COH). It is the task of clinicians to decide on suitable and cost-effective ovarian stimulation protocols for patients with a view to retrieving as many mature oocytes as possible, while also minimizing the risk of complications such as ovarian hyperstimulation syndrome (OHSS). This balance between efficacy and safety has been well-documented in the literature with the general consensus being that oocyte numbers in the low-to-mid teens provide a reasonable equilibrium for conventional COH protocols (van der Gaast *et al.*, 2006; Ji *et al.*, 2013; Magnusson *et al.*, 2018). Notwithstanding, a significant inter and even intra-individual variation has to be taken into account when addressing the issue of ovarian stimulation (Fauser *et al.*, 2008). Thus, clinicians are presented with the additional challenge of tailoring simulation protocols for patients which implicitly requires the prediction of how a specific patient will respond to a certain stimulation regimen at a certain time.

A number of works have attempted to predict the number of oocytes retrieved during simulation over the last 15 years (Fauser *et al.*, 2008). Many of these investigated the use of biological markers such as hormone levels (Dzik *et al.*, 2000; Majumder *et al.*, 2010; Jayaprakasan *et al.*, 2010; Oliveira *et al.*, 2012; Kummer *et al.*, 2013; Tulic *et al.*, 2020) and antral follicle count (AFC) (Ng *et al.*, 2000; Majumder *et al.*, 2010; Jayaprakasan *et al.*, 2010; Oliveira *et al.*, 2012). More recently, Barnett-Itzhaki *et al.* (2020) compared two machine learning (ML) models (an artificial neural network and a support vector machine) to a classical statistical modelling approach (logistic regression) to predict the number of oocytes retrieved from six covariates, finding that the former outperformed the latter. This reflects a wider trend in the fertility field in which techniques from ML have been successfully applied to a variety of problems from semen analysis (Hicks *et al.*, 2019) and blastocyst grading (Khosravi *et al.*, 2019) to providing prognoses to patients before treatment begins (Goyal *et al.*, 2020).

Behind many of these successes, however, lies the commitment of large datasets, a prerequisite for the use of many ML techniques (Rieke *et al.*, 2020). Obtaining such datasets poses a challenge to many researchers, especially in fertility, due to a fragmented data landscape in which datasets are closely guarded by their respective owners and data sharing is strictly regulated by local and international law. As a result, the process of accessing sensitive fertility data from external sources for research can prove convoluted and time-consuming, requiring months (or sometimes years) to complete (Hickman *et al.*, 2020).

In this work, we introduce and evaluate a new ML model for the prediction of oocytes retrieved during a stimulation cycle. This work is set apart from previous studies using ML in fertility by a key methodological innovation: though the models were fitted on third-party data from an external fertility clinic, no data transfer took place. Instead, a secure data collaboration framework was used to send the models to the external clinic, fit them and return the final models.

## Materials and methods

### Ethical approval

This study was approved by the local institutional review board committee. All participants gave written consent for the use of their data in retrospective scientific studies. All data were collected in accordance with the French National Commission for Information and Liberties.

### Study population, setting, and secure clinical data collection

This study was a retrospective analysis of a dataset of 11 286 uninterrupted autologous cycles performed at a single centre in France between 2009 and 2020. All cycles considered ended with a trigger shot. The dataset logged the values of 103 covariates pertaining to patient characteristics, treatment details, and outcomes (a full list can be found in the Supplementary Tables S1 and S2).

The starting dose of gonadotropins was individually adjusted according to the patients’ BMI, AMH level, AFC, and, if applicable, ovarian response to previous COH. Hormonal and ultrasound monitoring was performed in accordance with standard practice and recommendations throughout stimulation. When at least 3 follicles reached 17-mm diameter, ovulation was triggered with either recombinant HCG or GnRH agonist in case of significant OHSS risk. Egg collection was performed 36 h later. Estrogen (E2) priming was used in cycles involving antagonist protocols. All data was pseudonymized and strictly kept on the clinic’s server throughout the study.

**Figure 1.**
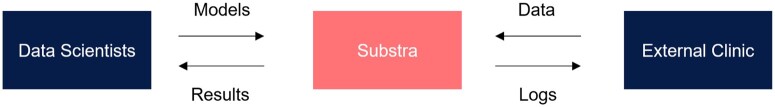
**Illustration depicting the study setting**.

Analysis of the data was carried out by a team external to the clinic. Having set up the necessary technical infrastructure, the analysis team sent source code for models to the clinic’s server using the Substra framework (Galtier and Marini, 2019). The code was executed on the clinic’s server and the results were sent back to the analysis team, the data (as well as logs from the execution of the code) always remaining on the clinic server. An illustration of this process can be seen in Fig. 1. Thus, the Substra framework provided a greater level of security than a more traditional approach involving a data transfer.

### Machine learning models

The prediction of the number of oocytes retrieved from a certain stimulation cycle was framed as a multivariate regression task. All analyses were carried out using Python 3.

### Feature/target selection

Of all the available covariates in the database, 16 features were selected according to their time of collection (all were collected before the start of the stimulation cycle) the magnitude of their Pearson correlation coefficients with respect to the number of oocytes retrieved, as well as input from clinicians. A list of these selected features can be found in Supplementary Table S1, a matrix of correlation coefficients is shown in Supplementary Fig. S1 and distribution plots in Supplementary Fig. S2. The available covariates that were not selected are shown in Supplementary Table S2.

The target variable was the number of oocytes retrieved for each cycle. A model was first trained to directly predict the target variable (0, 1, 2, 3, …). Moreover, and to better reflect clinical practice where ovarian response is considered a range rather than in precise numbers, additional models were trained to predict values of the target variable discretized into bins, arbitrarily provided by two clinicians according to their clinical experience. More specifically, these bins were {0, 1–3, 4–7, 8–12, 13–20, 21–29, 30+} for one clinician (who we shall henceforth refer to as Clinician A) and {0, 1–5, 6–10, 11–18, 19–25, 25+} for the other (henceforth Clinician B). The bins were then transformed into consecutive integer values (with, in the case of Clinician A, 0 becoming 0, 1–3 becoming 1, 4–7 becoming 2, and so on).

### Model and training details

Predictive models for each incarnation of the target variable were trained using the Light Gradient Boosting Machine (LightGBM) algorithm (Ke *et al.*, 2017). The LightGBM algorithm works by iteratively constructing an ensemble of decision trees. At inference time, the predictions of each tree in the ensemble are combined to provide a final prediction. An advantage of the LightGBM algorithm of particular use to our dataset is that it handles missing fields out-of-the-box. The models were trained using a training set that comprised 80% of the total included records. For the direct prediction model, the Poisson objective function was used; for the binned models the Huber loss was used. Hyperparameters (Supplementary Table S3) were determined through randomized search and 5-fold cross-validation optimizing the mean absolute percentage error (MAPE).

### Evaluation

The models were evaluated using the MAE and MAPE with respect to the ground truth on a held-out test set composed of the remaining 20% of the total included records. The model error distributions were plotted and compared.

Further analysis of the trained LightGBM models was carried out by estimating the amount of importance each model ascribed to each feature using the Shapley Additive Explanations (SHAP) framework (Lundberg and Lee, 2017), a method from the machine learning interpretability literature. To this end, mean absolute SHAP values for each feature were calculated for a random sample of 1000 patients passed through each model. Moreover, we compared our direct prediction model to a straightforward linear regression baseline model and our binned models to logistic regression baseline models. As these models do not take into account missing values, the mean of each variable across the dataset was used to fill in any missing values. The linear regression baseline was trained to convergence using the mean squared error objective whereas the logistic regression baselines were trained using the cross-entropy objective.

Finally, the behaviour of the model fitted to predict raw oocyte counts was analysed using a random sample of five patients. Each of the patient parameters was varied holding all others constant. The model’s predictions were plotted as a function of each individual parameter.

## Results

The median female age was 33 years [Q1–Q3: 30–36] and an antagonist protocol was used for the majority of patients (N = 10 253, 91.2%), with the remaining records being of agonist (N = 985, 8.5%) or unknown (N = 48, 0.4%) protocols. Further details on patient and treatment characteristics can be found in Tables 1 and 2.

**Table 1.** Patient characteristics for cycles in the dataset.

Characteristic		N	Median [Q1, Q3]
Age (years)		11 286	33 [30, 37]
Number of previous pregnancies		11 286	0 [0,1]
Type of infertility	Partner cause	4789	–
	Ovulatory disorder	5908	–
	Endometriosis	653	–
	Ovarian failure	1407	–
	Tubal disease	1290	–
	Other	719	–
WHO ovulatory disorder group	Normal	3053	–
	I	63	–
	II	6583	–
	III	751	–
Smoking status	Never smoked	7278	–
	Former smoker	2289	–
	Currently smoking	1705	–
BMI (kg/m^2^)		10 728	22.86 [20.44, 26.51]
Baseline AMH (µg/l)		8547	3.1 [1.9, 5.16]
Baseline FSH (IU/l)		9359	6.4 [5.3, 7.8]
Baseline E2 (pg/ml)		9191	37 [28, 49]
Baseline LH (IU/l)		9192	5.2 [3.9, 6.2]
Baseline testosterone (ng/ml)		7105	0.34 [0.23, 0.50]
Baseline TSH (IU/l)		7950	1.54 [1.10, 2.10]
Antral follicle count (2–9 mm)		5540	18 [12, 27]

**Table 2.** Treatment characteristics for cycles in the dataset.

Characteristic		N	Median [Q1, Q3]
Initial gonadotropin dose (IU)		11 183	225 [187.5, 300]
Protocol	Antagonist	10 253	–
	Agonist	985	–
Gonadotropin type	Gonal_F	4261	–
	Puregon	2819	–
	Menopur	3285	–
	Fostimon	100	–
	Pergoveris	175	
	Bemfola	82	
	Luveris	149	
	Fertistarkit	163	–
	Ovaleap	39	–
Number of oocytes retrieved		11 286	11 [7, 15]

### Model evaluation

**Figure 2.**
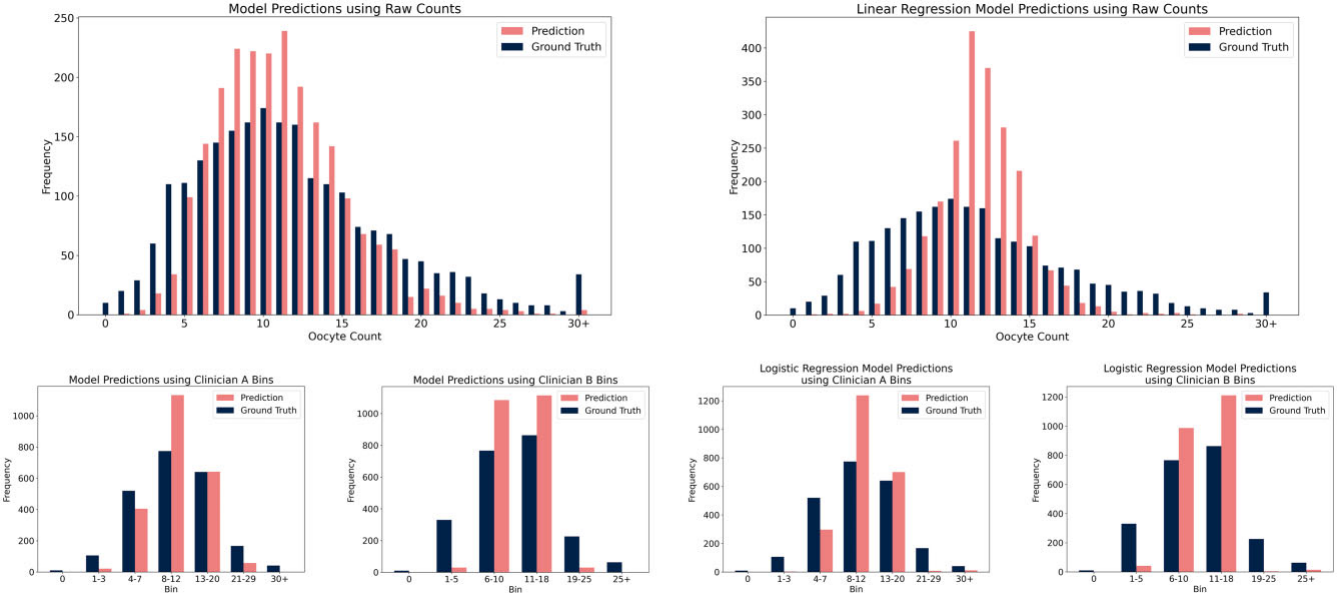
**Distribution of predictions by (from left to right, top to bottom) the direct prediction model, linear regression baseline, binned model for Clinician A, binned model for Clinician B, logistic regression baseline for Clinician A, and logistic regression baseline for Clinician B**.

On average, the model that directly predicted the number of oocytes retrieved deviated from the ground truth by 4.21 oocytes on average and achieved an MAPE of 0.52. The linear regression baseline deviated by an average of 4.36 oocytes with an MAPE of 0.63. The model for Clinician A deviated by an average of 0.73 bins with an MAPE of 0.29 whereas the logistic regression baseline deviated by an average of 0.77 bins with an MAPE of 0.32. The model for Clinician B deviated by 0.62 bins with an MAPE of 0.33 whereas the logistic regression baseline deviated by an average of 0.67 bins with an MAPE of 0.36. A comparison between the distributions of predictions by each model and the ground truth can be seen in Fig. 2a. An analogous comparison for the baseline models can be seen in Fig. 2b. Moreover, the performance of the models compared with baselines on the MAE and MAPE metrics can be seen in Table 3.

**Table 3.** Performance of models and baselines.

Target	Model	MAE	MAPE
Direct prediction	Ours	4.21	0.52
	Linear regression	4.36	0.63
Clinician A bins	Ours	0.73	0.29
	Logistic regression	0.77	0.32
Clinician B bins	Ours	0.62	0.33
	Logistic regression	0.67	0.36

MAE, mean absolute error; MAPE, mean absolute percentage error.

**Figure 3.**
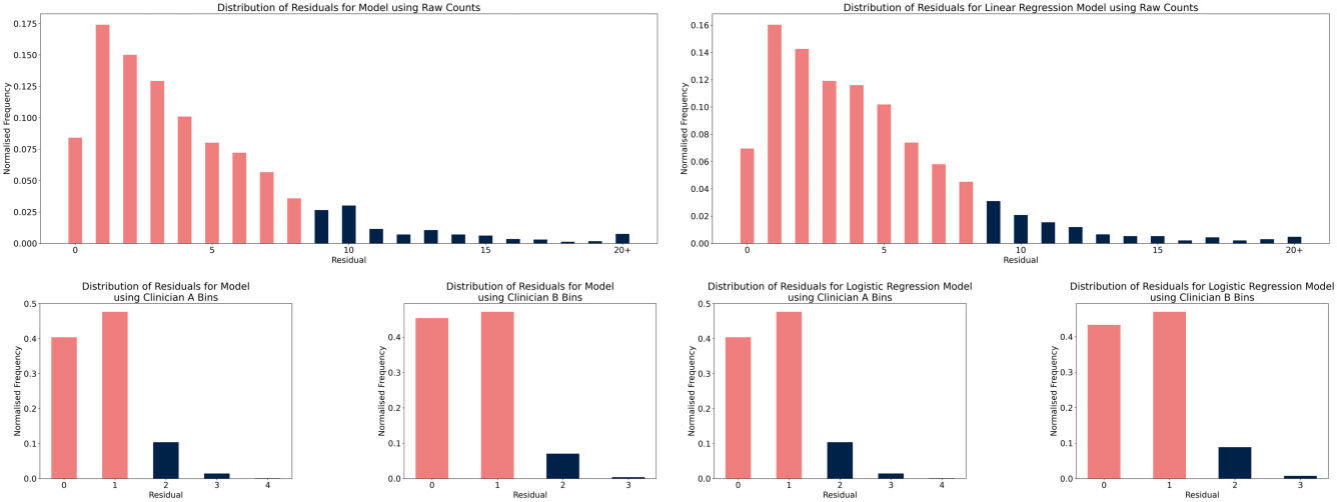
**Plots of the frequency with which each residual occurred for (from left to right, top to bottom) the direct prediction model, linear regression baseline, binned model for Clinician A, binned model for Clinician B, logistic regression baseline for Clinician A, and logistic regression baseline for Clinician B.** The bars that encompass the first 85% of predictions for each model are highlighted in pink.

Overall, the LightGBM models captured the distributions quite well, with the majority of records within the first and third quartiles being predicted correctly within 2 oocytes or 1 clinician-defined bin (53% for the direct prediction model compared to 64% for the baseline; 53% for the both the model with Clinician A’s bins and the baseline; 61% for the model with Clinician B’s bins and 59% for the baseline). However, the models did not seem to capture the tails of the distributions well, underpredicting the number of zero-oocyte cycles as well as 15+ oocyte cycles (which comprised 22% of the true distribution but only 11% of the LightGBM predicted distribution and 7% for the baseline predicted distribution). Nevertheless, 41.5% of the records in the first quartile were predicted correctly within 2 oocytes by the direct prediction LightGBM model compared to 12% for the baseline model. This is illustrated in Fig. 3a and b by plotting the frequency distribution of residuals for each model. We further plotted the mean residuals of predictions relative to their true values (Supplementary Figure S3). For the direct prediction model, 88% of predictions were correct within seven oocytes; the binned models both achieved over 85% of predictions being within one bin of the correct bin.

**Figure 4.**
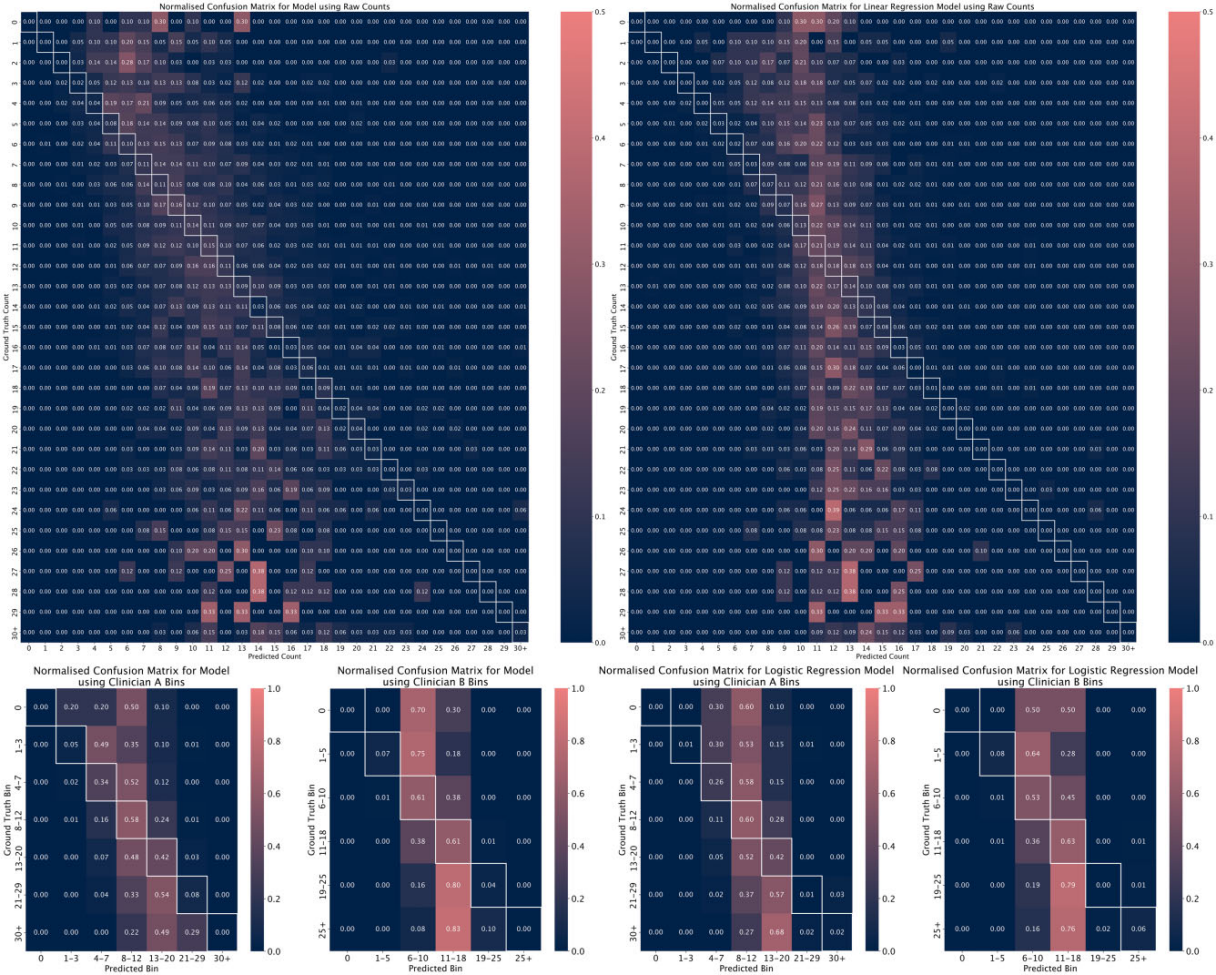
**Normalized confusion matrices for (from left to right, top to bottom) the direct prediction model, linear regression baseline, binned model for Clinician A, binned model for Clinician B, logistic regression baseline for Clinician A, and logistic regression baseline for Clinician B.** We highlight the cells on the diagonal of each matrix for ease of interpretation.

Finally, confusion matrices were computed for each model (Fig. 4a and b), showing in detail the predictions made by the models relative to the ground truth. The columns of the matrices are normalized to sum up to 1. The predictions made by the model for the absolute number of oocytes tend towards fair association with the true value—especially when the number of oocytes is between 4 and 15. When the target value was binned, the resulting models performed well, with almost all predictions falling within one bin of the ground truth (88% with Clinician A’s bins; 92% with Clinician B’s bins).

### Model interpretation

**Figure 5.**
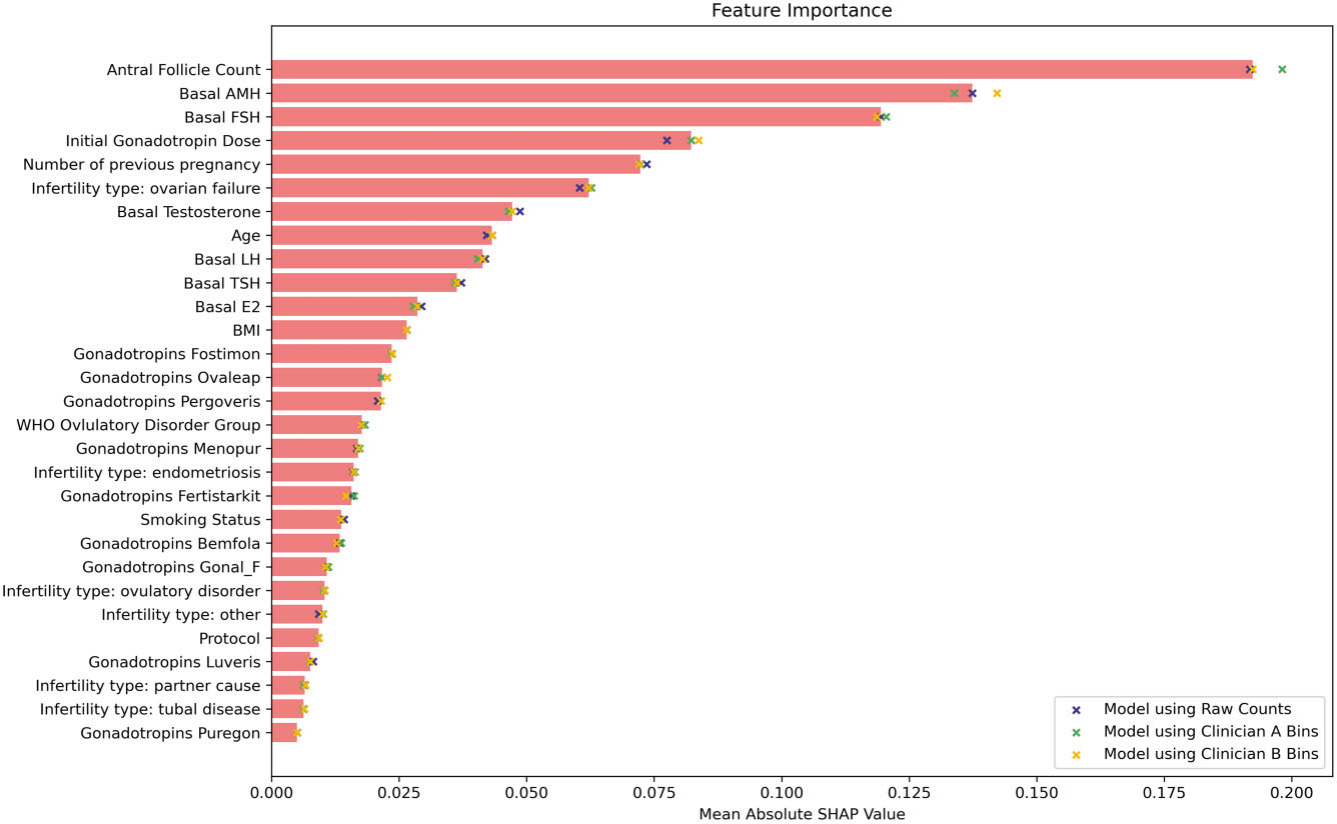
**A plot of each feature’s mean absolute Shapley Additive Explanation (SHAP) value across the three models**. The solid bars give the median mean absolute SHAP values; the crosses denote the mean absolute SHAP value calculated for each model individually. The higher the SHAP value, the more important the feature.

Overall, all three models agreed on the importance of each feature. The feature with the highest mean absolute SHAP value (and thus the highest importance) was the AFC, followed by basal AMH, FSH, and initial gonadotropin dose. SHAP values for all features are seen in Fig. 5.

**Figure 6.**
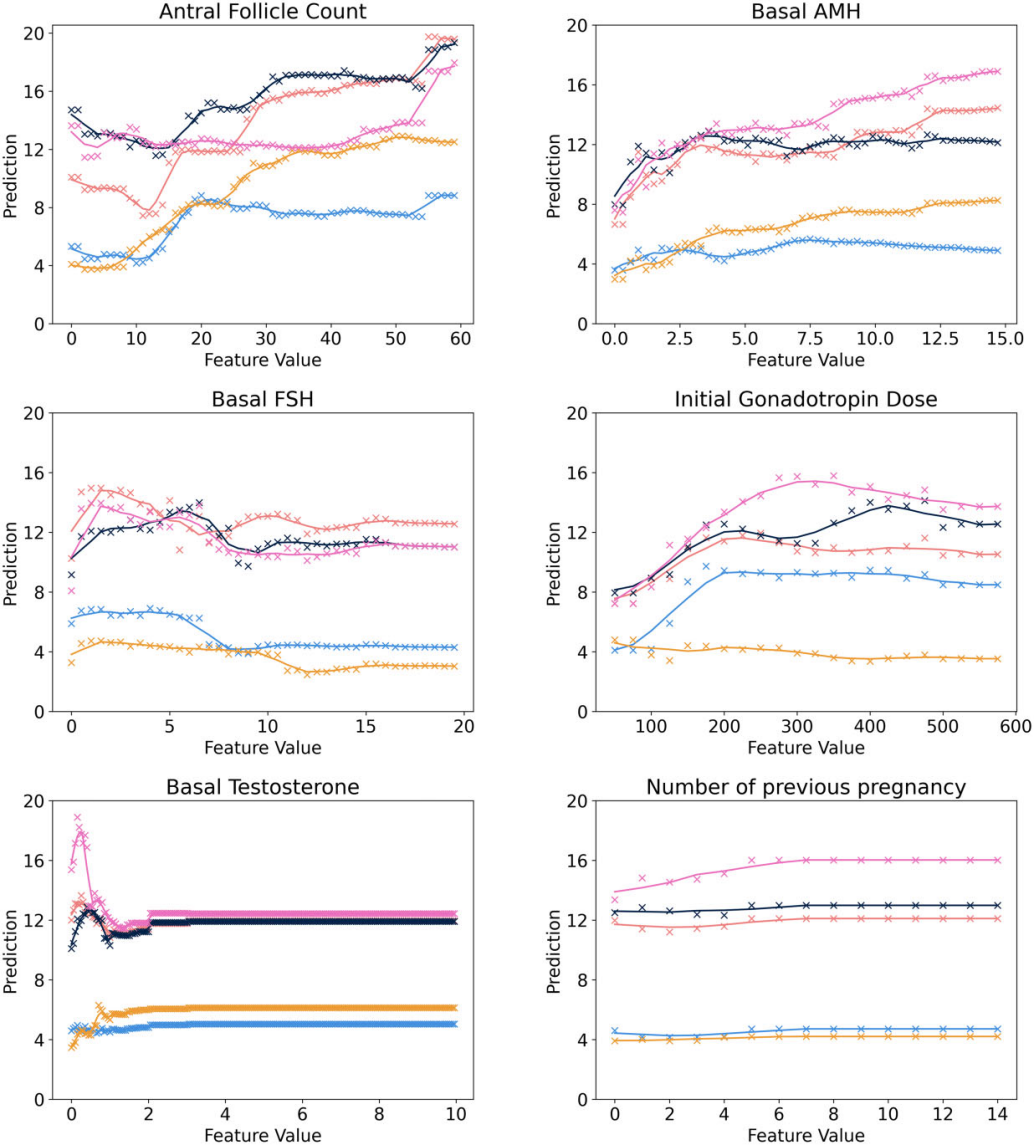
**Plots of model predictions as a function of the six variables with the highest Shapley Additive Explanation (SHAP) values for 5 patients.** Each colour represents a different patient. We exclude categorical variables. Plots of the other variables can be found in Supplementary Fig. S4.

Plotting the predictions made by the model trained to predict the raw number of oocytes retrieved revealed it to have picked up a number of correlations (see Fig. 6 and Supplementary Fig. S4). As expected, the model identified an upward trend between the number of oocytes retrieved and increasing AFC and basal AMH. The plot for the initial dose of gonadotropin exhibited a ‘bump’ shape: the predicted number of oocytes increased until a certain dose is reached considering a patient prognosis and then plateaued.

## Discussion

In this study, we demonstrated that machine learning models could be securely and successfully trained to predict the number of oocytes retrieved from COH.

### Analysis of model evaluation

Our results demonstrate that the fitted models were able to predict the number of oocytes retrieved from COH for most records within the range of a single clinician-defined bin. For all models, performance was best within the second and third quartiles of the target variable, with the model underpredicting extreme values of the target variable (no oocytes and large numbers of oocytes retrieved). Nevertheless, the confusion matrices illustrated that the erroneous predictions made cycles leading to a small number of oocytes were still within the vicinity of the true value and not entirely incorrect. There were almost no cases in which a patient with a very large number of oocytes retrieved was predicted to have fewer than three oocytes or vice versa.

In addition, the LightGBM models not only performed better than the baseline models in terms of the MAE and MAPE metrics but also better captured the overall distribution of the target variable as can be seen in Fig. 2a and b. There were also more accurate in predicting the first quartiles as reflected in the MAPE scores. On the contrary, predictions from the baseline models seem centred around the dataset mean which led to good performance on average, but huge underprediction of the distribution tails. These results highlight the importance of looking beyond just single, quantitative, one-number metrics when evaluating the performance of a model.

Furthermore, in the case of the upper extrema (>20 oocytes), it may not be necessary from a clinical standpoint to accurately predict the exact number of oocytes retrieved. This is due to the fact that any number of oocytes in the upper extremum would be more than sufficient to proceed with treatment (Ji *et al.*, 2013) and is reflected in the clinician-provided bins. Notwithstanding, such a prediction would also suggest that the proposed COH protocol may pose a risk of OHSS to the patient and that a milder protocol ought to be considered (Fauser *et al.*, 2010).

A key advantage of this study over Barnett-Itzhaki *et al.* (2020) is that we treat the prediction of oocytes retrieved from COH as a regression problem over the target variable as well as clinician-provided bins. Barnett-Itzhaki *et al.* (2020), on the other hand, formulated the task as a classification problem over tertiles of the target variable. Though such an approach actually works, treating the number of oocytes retrieved as a purely categorical variable disregards the ordinal relation between the categories. That is, that misclassifying a no-oocyte cycle as a 5-oocyte cycle is ‘less wrong’ than misclassifying it as a 20-oocyte cycle. Furthermore, the prediction of tertiles is not as clinically relevant as direct prediction of the number of oocytes retrieved or prediction of clinician-provided bins.

Another advantage of our study is that it considered a far larger dataset (by a factor of approximately 100, made possible by the Substra framework). Despite this, our study is still limited in that it only considered a single-clinic dataset thus likely not representing the entire patient population. However, our results provide a strong case for a follow-up study expanding to an international multi-clinic setting through the Substra framework. In addition, further evaluation of the models’ predictions with respect to those of human clinician, perhaps in a prospective setting, should be considered before they can be translated into clinical practice to assist clinicians in optimizing COH protocols.

### Analysis of model interpretation

From Fig. 5, there is a clear consensus among the three models over the ranking of features in terms of their SHAP values (a proxy for their ‘importance’). The SHAP values suggest that the most important hormonal marker was basal AMH. This was followed by basal FSH. These findings corroborate widespread clinical practice (Tehraninezhad *et al.*, 2016). Basal testosterone, LH, TSH and E2 were less important. Nevertheless, Blom *et al.* (2018) found that serum E2 levels on the fifth day of stimulation were significantly associated with serum E2 levels on the day of triggering, a strong predictor of stimulation response (Kummer *et al.*, 2013)—future work might be able to achieve greater clinical value by recording and considering such during-treatment measurements on top of baseline characteristics.

Of the non-hormonal patient characteristics, AFC had the highest SHAP value. This finding is not too surprising, given the large body of previous work highlighting the association between AFC and ovarian response (Ng *et al.*, 2000; Majumder *et al.*, 2010; Jayaprakasan *et al.*, 2010; Oliveira *et al.*, 2012). The patient’s medical history (infertility types) and the number of previous pregnancies (cycle rank) were also deemed important by the models. Such a trend is well-documented in the literature (Paul *et al.*, 2020; Tarín *et al.*, 2020).

Lastly, the models were not able to detect whether a patient has a specific affinity for a type of gonadotropin. Indeed, there is currently little consensus on the type of gonadotropins to use (Bergandi *et al.*, 2020).

### Evaluation of the Substra framework for secure data management

Though training models through the Substra framework was smooth, a key limitation was encountered during the course of this study. Evaluation of the models proved difficult to do as the Substra framework only allows results to be sent in the form of a single number. Though sufficient for sending over the metrics such as testing accuracy, this restriction posed a problem when trying to analyse the distribution of prediction errors. In the end, the analysis team had to ask the centre to run the code that calculated the error distributions and manually return the results. Such a problem, however, could have been mitigated if the analysis team had access to their own small dataset for performing such evaluations.

It is also important to note that, to set up the study, an investment of time and capital was also required. In particular, the data analysis team spent a significant length of time learning to use the Substra framework and a new server had to be installed by the external clinic to host and run models. Moreover, a great deal of communication between the clinic and data analysis team was required to resolve technological problems during this set-up period. Such collaboration is especially important to ensure the quality of the data made available through the framework: as the data analysis team does not have access to the data, it is up to the clinic to ensure the validity of the data they supply to researchers. Nevertheless, once the investment in time and capital have been made, the availability of the no-transfer strategy has the potential to simplify future research collaborations greatly, by eliminating some of the legal burdens associated with traditional data transfers. Moreover, if adopted at a wider level, the use of no-transfer frameworks may help simplify and standardize the collection of larger, multi-centre datasets.

## Conclusion

In this study, we have presented new predictive models for the number of oocytes retrieved following controlled ovarian hyperstimulation. These models have the potential to form a basis for future clinical decision-making tools. The models were fitted using data from an external clinic without the need for an inter-clinic data transfer, guaranteeing total security. Our work thus demonstrates the promise of using the Substra framework for allowing external researchers to provide clinically relevant insights on sensitive fertility data in a secure, trustworthy manner and opens a number of exciting avenues for accelerating future research in personalized medicine.

## Supplementary Material

dead163_Supplementary_Figure_S1

dead163_Supplementary_Figure_S2

dead163_Supplementary_Figure_S3

dead163_Supplementary_Figure_S4

dead163_Supplementary_Table_S1

dead163_Supplementary_Table_S2

dead163_Supplementary_Table_S3

## Data Availability

The data underlying this article cannot be shared publicly for the privacy of individuals that participated in the study. The data will be shared on reasonable request to Thomas Fréour (thomas.freour@chu-nantes.fr). Approval of a proposal will be necessary before data will be shared. To gain access, requesters will need to sign an agreement form and confirm that the data will be used for the purpose for which access was granted.
