# Supplementary material for: Predicting the number of oocytes retrieved from controlled ovarian hyperstimulation with machine learning
Source: Hum Reprod. 2023 Aug 15;38(10):1918–26. doi: 10.1093/humrep/dead163 (PMC10546073; doi:10.1093/humrep/dead163)
Supplement: dead163_Supplementary_Figure_S1 [file dead163_supplementary_figure_s1.pdf]

OXFORD
human reproduction
Human Reproduction, 2023, 1–1
https://doi.org/10.1093/humrep/dead163
Supplementary Data

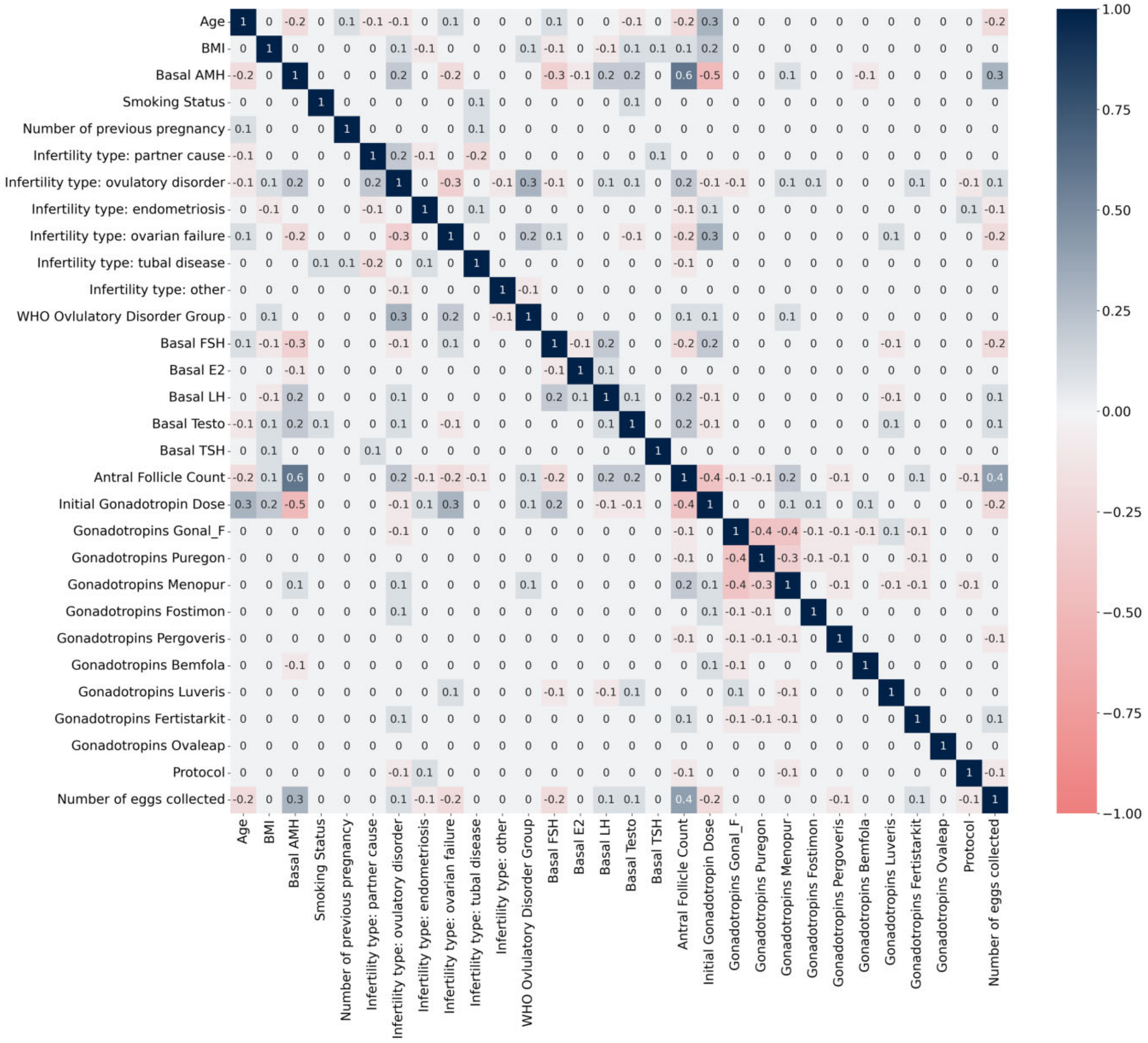

Supplementary Figure S1. Spearman correlation coefficients between parameters in the dataset.
