# Supplementary material for: Predicting the number of oocytes retrieved from controlled ovarian hyperstimulation with machine learning
Source: Hum Reprod. 2023 Aug 15;38(10):1918–26. doi: 10.1093/humrep/dead163 (PMC10546073; doi:10.1093/humrep/dead163)
Supplement: dead163_Supplementary_Figure_S3 [file dead163_supplementary_figure_s3.pdf]

OXFORD
human reproduction
Human Reproduction, 2023, 1–1
https://doi.org/10.1093/humrep/dead163
Supplementary Data

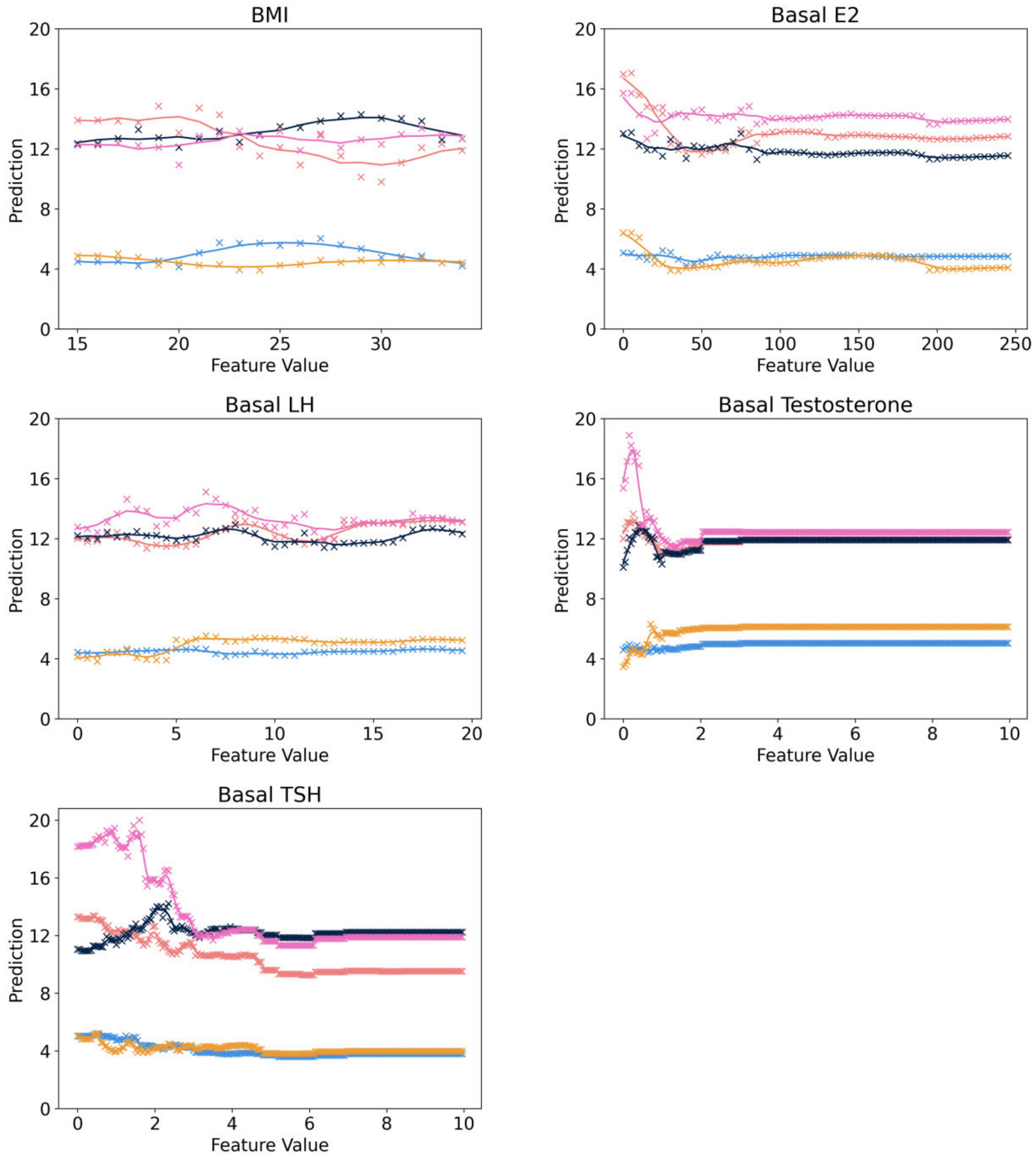

Supplementary Figure S3. Plots of average residuals for each ground truth value for (from left to right, top to bottom) the direct prediction model, linear regression baseline, binned model for Clinician A, binned model for Clinician B, logistic regression baseline for Clinician A, and logistic regression baseline for Clinician B.
