# Supplementary material for: Predicting the number of oocytes retrieved from controlled ovarian hyperstimulation with machine learning
Source: Hum Reprod. 2023 Aug 15;38(10):1918–26. doi: 10.1093/humrep/dead163 (PMC10546073; doi:10.1093/humrep/dead163)
Supplement: dead163_Supplementary_Table_S1 [file dead163_supplementary_table_s1.pdf]

OXFORD
human reproduction

Human Reproduction, 2023, 1–1
https://doi.org/10.1093/humrep/dead163
Supplementary Data

Supplementary Table S1. Features used in the models.

Feature	Definition
Age	Age of patient at start of treatment.
Basal AMH	Anti-Mullerian Hormone level at baseline. Measured in ng/ml.
BMI	Patient's body mass index.
Initial Gonadotropin Dose	Initial dose of gonadotropin. Measured in IU.
E2 basal levels	Estradiol levels at start of treatment. Measured in pg/ml.
AFC	Patient's antral follicle count.
Basal FSH	Follicle-stimulating hormone levels at baseline. Measured in mUI/ml.
Infertility type: partner cause	Does the couple have a cause of infertility due to the partner?
Infertility type: ovulatory disorder	Does the patient have the cause of infertility ovulatory disorder?
Infertility type: endometriosis	Does the patient have the cause of infertility endometriosis?
Infertility type: ovarian failure	Does the patient have the cause of infertility ovarian failure?
Infertility type: tubal disease	Does the patient have the cause of infertility tubal disease?
Infertility type: other	Does the patient have a cause of infertility classified as other?
Basal LH	Luteinizing Hormone levels at baseline. Measured in mUI/ml.
Number of previous pregnancies	Number of previous pregnancies.
number of oocytes retrieved	Number of collected oocytes.
Protocol	Stimulation protocol—could be agonist or antagonist.
Gonadotropins Gonal-F	Whether Gonal-F was used during stimulation or not.
Gonadotropins Puregon	Whether Puregon was used for stimulation or not.
Gonadotropins Menopur	Whether Menopur was used for stimulation or not.
Gonadotropins Fostimon	Whether Fostimon was used for stimulation or not.
Gonadotropins Pergoveris	Whether Pergoveris was used for stimulation or not.
Gonadotropins Bemfola	Whether Bemfola was used for stimulation or not.
Gonadotropins Luveris	Whether Luveris was used for stimulation or not.
Gonadotropins Fertistarkit	Whether Fertistarkit was used for stimulation or not.
Gonadotropins Ovaleap	Whether Ovaleap was used for stimulation or not.
WHO ovulatory disorder status	World Health Organisation ovulatory disorder status.
Smoking status	One of: never smoked, former smoker or smoker.
Basal testosterone	Testosterone levels at baseline.
Basal TSH	TSH levels at baseline.
