# Supplementary material for: Predicting the number of oocytes retrieved from controlled ovarian hyperstimulation with machine learning
Source: Hum Reprod. 2023 Aug 15;38(10):1918–26. doi: 10.1093/humrep/dead163 (PMC10546073; doi:10.1093/humrep/dead163)
Supplement: dead163_Supplementary_Table_S2 [file dead163_supplementary_table_s2.pdf]

OXFORD
human reproduction

Human Reproduction, 2023, 1–2
https://doi.org/10.1093/humrep/dead163
Supplementary Data

Supplementary Table S2. Features not used in the models.

Feature	Definition
Year of treatment	Year in which the treatment was performed.
Cancellation reason	Cancellation reason of a cycle.
Last measure of endometrial thickness	Last measure of the endometrium during stimulation. Measured in mm.
Gonadotropin dose between Days 1 and 6	The total gonadotropin dose administered in the first 6 days of stimulation. Measured in IU.
Total gonadotropin dose	Total dose of gonadotropin throughout the stimulation. Measured in IU.
Duration of gonadotropin treatment	Number of days of gonadotropin treatment.
E2 on blocking day	Estradiol levels on estradiol priming day. Measured in pg/ml.
E2 on stimulation Day 1	Estradiol levels at Day 1 of stimulation. Measured in pg/ml.
E2 on stimulation Day 5	Estradiol levels at Day 5 of stimulation. Measured in pg/ml.
E2 on stimulation Day 6	Estradiol levels at Day 6 of stimulation. Measured in pg/ml.
E2 on stimulation Day 7	Estradiol levels at Day 7 of stimulation. Measured in pg/ml.
E2 on stimulation Day 8	Estradiol levels at Day 8 of stimulation. Measured in pg/ml.
E2 on trigger	Last E2 levels assessment during stimulation (typically within a day of triggering). Measured in pg/ml.
E2 on trigger Day +1	Estradiol levels the day after the trigger. Measured in pg/ml.
E2 on trigger Day +2	Estradiol levels 2 days after the trigger. Measured in pg/ml.
E2 on trigger day	Estradiol levels at trigger. Measured in pg/ml.
Endometrial thickness on trigger day +1	Endometrium measurement on the first day after trigger. Measured in mm.
Endometrial thickness on trigger day	Endometrium measurement on the trigger day. Measured in mm.
pregnancy status	Has the treatment lead to pregnancy?
Infertility type	Reason for performing the treatment.
Partner infertility	Type of infertility for the partner.
Infertility categorization	Primary of secondary infertility type.
Main infertility reason	Main infertility reason.
Transfer day	Day of embryo transfer.
Trigger day	Number of stimulation days before triggering.
Last blood test day	Day where the patient had her last blood test.
Retrieval day	Number of days before egg collection.
LH on blocking day	LH levels at Day 0 of stimulation. Measured in mUI/mL.
LH on stimulation Day 1	LH levels at Day 1 of stimulation. Measured in mUI/mL.
LH on stimulation Day 5	LH levels at Day 5 of stimulation. Measured in mUI/mL.
LH on stimulation Day 6	LH levels at Day 6 of stimulation. Measured in mUI/ml.
LH on stimulation Day 7	LH levels at Day 7 of stimulation. Measured in mUI/ml.
LH on stimulation Day 8	LH levels at Day 8 of stimulation. Measured in mUI/ml.
LH on last check	LH levels at last blood test during stimulation. Measured in mUI/ml.
LH on trigger Day +1	LH levels the day after the trigger. Measured in mUI/ml.
LH on trigger Day +2	LH levels two days after the trigger. Measured in mUI/ml.
LH on trigger day	LH levels on the day of the trigger. Measured in mUI/ml.
Number of cycles	Number of assisted reproductive technology cycles undergone by the patient (including the current one).
Number of previous deliveries	Number of previous deliveries.
Partner number of cigarettes per day	Partner number of cigarettes per day.
Number of cigarettes per day	Patient-reported number of cigarettes per day.
Number of previous spontaneous miscarriage	Number of previous spontaneous miscarriage.
number of oocytes injected	Number of oocytes to ICSI.
number of oocytes inseminated	Number of oocytes to IVF.
2PN oocytes on Day 1	Number of 2PN oocytes at Day 1.
3PN oocytes on Day 1	Number of 3 + PN oocytes at Day 1.
Number of lysed oocytes	Number of lysed oocytes.
number of oocytes selected	Number of retained oocytes.
Number of mature oocytes	Number of mature oocytes.
Number of mature oocytes on trigger day	Number of mature oocytes on trigger day.
Partner weight	Weight of the partner.
Patient weight	Weight of the patient.
Progesterone on blocking day	Prog. levels at stimulation Day 0. Measured in ng/ml.
Progesterone on Day 1	Prog. levels at stimulation Day 1. Measured in ng/ml.
Progesterone on Day 5	Prog. levels at stimulation Day 5. Measured in ng/ml.
Progesterone on Day 6	Prog. levels at stimulation Day 6. Measured in ng/ml.
Progesterone on Day 7	Prog. levels at stimulation Day 7. Measured in ng/ml.
Progesterone on Day 8	Prog. levels at stimulation Day 8. Measured in ng/ml.
Progesterone on last check	Prog. levels at the last blood test. Measured in ng/ml.
Progesterone on trigger Day −1	Progesterone levels the day after the trigger. Measured in ng/ml.
Progesterone on trigger Day −2	Progesterone levels two days after the trigger. Measured in ng/ml.

(continued)

Supplementary Table S2. (continued)

Feature	Definition
Progesterone on trigger day	Progesterone levels at trigger day. Measured in mUI/mL.
Cycle rank	One more than the number of previous pregnancies for the patient.
Embryonic reduction	Was there any embryo reduction during the cycle?
Endometriosis status	Level of endometriosis.
Partner smoking status	One of: never smoked, former smoker, or smoker.
Fertilization rate for IVF	IVF fecondation percentage rate.
Fertilization rate for ICSI	ICSI fertilization fecondation percentage rate.
Treatment type	One of: IVF, ICSI, IVF + ICSI.
Treatment stop	One of: cancelled, triggered.
Trigger type	One of: agonist or HCG.
Gonadotropins type	Daily gonadotropins or Elonva.
Uterus information	Relevant indication about the uterus of the patient.
