# Supplementary material for: Predicting the number of oocytes retrieved from controlled ovarian hyperstimulation with machine learning
Source: Hum Reprod. 2023 Aug 15;38(10):1918–26. doi: 10.1093/humrep/dead163 (PMC10546073; doi:10.1093/humrep/dead163)
Supplement: dead163_Supplementary_Table_S3 [file dead163_supplementary_table_s3.pdf]

OXFORD
human reproduction

Human Reproduction, 2023, 1–1
https://doi.org/10.1093/humrep/dead163
Supplementary Data

Supplementary Table S3. Hyperparameters used for training each model.

	Value		
**Hyperparameter**	**Raw counts model**	**Clinician A model**	**Clinician B model**
Boosting type	GBDT	GBDT	GBDT
Importance type	Split	Split	Split
Learning rate	0.1	0.05	0.05
Column sample for each tree	0.95	0.75	0.95
Maximum depth	15	90	N/A
Minimum samples per leaf	10	40	25
Minimum sum of instance weight per leaf	0.001	0.001	0.001
Minimum split gain	0	0	0
Number of trees	1000	600	600
Number of leaves	220	300	64
L1 regularization weight	0	0	0
